# EFFECTS OF HIGH-DOSE VITAMIN C AND HYALURONIC ACID ON TENDON HEALING

**DOI:** 10.1590/1413-785220182602182353

**Published:** 2018

**Authors:** YASAR MAHSUT DINCEL, OKTAY ADANIR, YAVUZ ARIKAN, AYSEL KARA CAGLAR, SUZAN CANSEL DOGRU, YUNUS ZIYA ARSLAN

**Affiliations:** 1. Department of Orthopedics and Traumatology, Metin Sabanci Baltalimani Bone Diseases Training and Research Hospital, Istanbul, Turkey.; 2. Department of Orthopedics and Traumatology, Istanbul Bagcilar Training and Research Hospital, Istanbul, Turkey.; 3. Department of Medical Pathology, Istanbul Bagcilar Training and Research Hospital, Istanbul, Turkey.; 4. Department of Mechanical Engineering, Faculty of Engineering, Istanbul University, Avcilar, Istanbul, Turkey.

**Keywords:** Hyaluronic acid, Vitamin C, Rats, Achilles tendon, Ácido hialurônico, Vitamina C, Ratos, Tendão do calcâneo.

## Abstract

**Objective:**

To assess the histopathologic and biomechanical effects of hyaluronic acid (HA) and high-dose vitamin C (VC) on rat Achilles tendon healing.

**Methods:**

Forty-eight Sprague-Dawley rats were randomized to HA and VC and control groups with equal numbers. Each group was further divided into two subgroups to be sacrificed on Day 15 (n=8) and Day 30 (n=8). The Achilles tendons were cut and repaired. While the control rats remained untreated, HA and VC were administered after repair. The repaired tendons were removed for biomechanical and histopathologic analyses. In the biomechanical tests, the tendons were stretched to failure and maximum forces were measured. For histopathologic examination, the specimens were interpreted semiquantitatively using Movin’s grading scale and Bonar scores.

**Results:**

The highest mean forces were obtained in the HA group on Day 15 and in the VC group on Day 30, with a significant difference between HA and VC on Day 15 between control and VC on Day 30 (p<0.05). Histological examination showed both Movin and Bonar scores decreased in all groups on Day 30, with significant improvements in the HA and VC groups (p<0.05).

**Conclusion:**

Our results demonstrated that both VC and HA had therapeutic effects on tendon healing, especially in the late phase. Level of Evidence I; High quality randomized trial with statistically significant difference.

## RESUMO

**Objetivo:**

Avaliar os efeitos histopatológicos e biomecânicos do ácido hialurônico (AH) e altas doses de vitamina C (VC) na cicatrização do tendão do calcâneo em ratos.

**Métodos:**

Quarenta e oito ratos Sprague-Dawley foram randomizados em grupo AH, grupo VC e grupo controle, iguais em número. Cada grupo foi dividido em dois subgrupos a serem sacrificados no Dia 15 (n = 8) e no Dia 30 (n = 8). Os tendões do calcâneo foram cortados e reparados. Enquanto os ratos do grupo controle permaneceram não tratados, os do grupo AH/VC receberam AH e VC após o reparo. Os tendões reparados foram removidos para análises biomecânicas e histopatológicas. Nos testes biomecânicos, os tendões foram estirados até a falha e as forças máximas foram medidas. No exame histopatológico, as amostras foram interpretadas semiquantitativamente usando os escores de Movin e de Bonar.

**Resultados:**

As forças médias mais altas foram obtidas no grupo AH no Dia 15 e no grupo VC, no Dia 30, com diferença significativa entre os grupos HA e VC no Dia 15 e entre os grupos controle e VC (p < 0,05). No exame histológico, os escores de Movin e Bonar diminuíram em todos os grupos no dia 30, com melhora significativas nos grupos AH e VC (p < 0,05).

**Conclusões:**

Nossos resultados demonstraram que tanto a VC quanto o AH tiveram efeitos terapêuticos sobre cicatrização do tendão, especialmente na fase tardia. Nível de Evidência I; Estudo clínico randomizado de alta qualidade com ou sem diferença estatisticamente significante.

## INTRODUCTION

Achilles tendon rupture is typically seen in men during their thirties and forties who rarely engage in sports and spend most of their time in office work.^1^ Researchers have examined many factors to better understand the mechanisms of tendon healing to speed this process. These factors include growth factors, mesenchymal stem cells, cytokines, gene therapy approaches, sodium hyaluronate, platelet concentrates, anticoagulants, and hyperbaric oxygen.^2^ ^,^ ^3^ However, there is no gold-standard treatment that can improve tendon healing by applying exogenous agents.

Hyaluronic acid (HA) is known to have a preventive effect on adhesions, but its effects on tendon biomechanics are not fully known. Although HA has been used in humans after tendon repair, consensus on its benefits is lacking. Meanwhile, vitamin C (VC) has been shown to have beneficial effects on tendon healing, such as increased collagen fibril diameter, promotion of angiogenesis, and increased number of fibroblasts in the healing period.^4^ However, the number of studies regarding the effects of VC on tendon healing is limited.

Most of the previous studies focusing on the effects of HA and VC in tendon healing have included either histopathological or biomechanical results, but not both.^3^ ^-^ ^6^ A few recent studies focusing on the therapeutic effects of HA have provided both histopathological and biomechanical results.^7^ Furthermore, some studies (especially on HA) have examined the effects of these two drugs on adhesiveness of tendons rather than on tendon healing.^8^ In this regard, the purpose of this experimental study was to investigate the histopathological, pathophysiological, and biomechanical results of applying HA and VC to Achilles tendon ruptures treated with primary repair. 

## MATERIALS AND METHODS

The study was approved by the local institutional ethics review board (approval number: 2014/22). A total of 48 adult male 7-month-old Sprague-Dawley rats weighing 300-350 g were used. After randomization, two study groups were formed to receive hyaluronic acid (HA group: *n*=16) and high-dose vitamin C (VC group: *n*=16), and the remaining 16 rats were assigned as the control group. In each group, the rats were further divided into two equal subgroups to be sacrificed on Day 15 and Day 30.

The rats were placed in the supine position on the operating table and received inhalation anesthesia. The site of the operation was disinfected using a povidone-iodine cleansing solution (Betadine, Kansuke, Istanbul, Turkey). A straight skin incision was made on the left side starting 1 cm proximal to the calcaneal insertion of the Achilles tendon and extending in a longitudinal proximal direction 2-3 cm toward the caudal surface. After excision of the subcutaneous connective tissue and tendon sheath, the Achilles tendon was exposed. In all the rats, the left Achilles tendons were cut transversely 4-5 mm proximal to the calcaneal insertion with a number 11 scalpel blade (Plusmed, Turkey) and repaired using the modified Kessler technique with a PDO II 4/0 suture (BOZ, Ankara, Turkey). In the HA group, 0.075 ml/kg HA (Hyaloglide) was administered via a micropipette on the site of the tendon repair. The wound site was closed with 3/0 prolene (Dogsan, Istanbul, Turkey) and covered with a sterile dressing after application of povidone-iodine (Betadine, Kansuke, Istanbul, Turkey). High-dose vitamin C (Vitabiol C) was administered intraperitoneally with an insulin syringe on alternate days, starting after skin closure until sacrifice. The control rats received no medication after primary tendon repair. 

None of the rats were immobilized postoperatively. Eight rats in each group were sacrificed with high-dose anesthesia on Day 15 and Day 30. The repaired Achilles tendons were removed with excision of the calcaneal and femoral condyle insertions. Of the eight repaired Achilles tendons in each subgroup, five samples were used for biomechanical measurement and three for histopathologic examination. To eliminate any confounding effect on biomechanical measurements, care was taken to not remove the plantaris tendon during removal of the Achilles tendon. All the samples were taken to the biomechanical laboratory on the day of removal in sterile containers. For histopathologic examination, each sample was placed in a sterile 10% formaldehyde solution and sent to our pathology clinic on the same day.

### Biomechanical tests

To mount the tendon specimens onto the tensile test machine, tendon-muscle and tendon-bone regions were securely fixed between sandpaper sheets, which were then attached to the grips of the testing machine. The tendon specimens were stretched to failure along their long axis at a constant speed of 6 mm/min. During the tensile test, data on the tensile force were recorded and maximum force that led to tendon rupture was determined.^9^

### Histopathologic study

All tendon specimens were fixed in 20 ml of 10% formalin for 24 hours, placed in tissue cassettes and then in closed sample-tracking equipment for processing in alcohol, acetone, xylene, and paraffin for dehydration. Paraffin blocks were then obtained and sectioned at 4µm. Sections were stained with hematoxylin-eosin (H-E), Masson’s trichrome (MT), and alcian blue at pH 2.5. Stained slides were covered with a drop of fixing solution and closed with a lamella. 

The slides were interpreted semiquantitatively using Movin’s grading scale and Bonar’s scores.^10^

For Movin’s grading scale, eight variables were examined, which included (1) fiber structure, (2) fiber arrangement, (3) rounding of the nuclei, (4) regional variations in cellularity, (5) increased vascularity, (6) decreased collagen stainability, (7) hyalinization, and (8) glycosaminoglycan (GAG) content. The first seven variables were assessed on the H-E stained slides and the GAG content was assessed on the alcian blue-stained slides. Each variable was graded between 0 and 3: normal (0), slightly abnormal (1), abnormal (2), and markedly abnormal (3). Total semiquantitative histologic scores varied between 0(normal tendon) and 24 (the most severe abnormality).

Four variables were scored in the Bonar system: (1) tenocytes, (2) ground substance, (3) collagen, and(4) vascularity. A four-point scoring system was used, from 0, indicating a normal appearance, to 3, indicating a markedly abnormal appearance. For each assessment, the total score varied between 0(normal tendon) and 12(most severe abnormality).

### Statistical analysis

For descriptive statistics, data were expressed as mean, standard deviation, median, and the minimum value. Distribution of the variables was measured by the Kolmogorov-Smirnov test. Histopathologic findings were analyzed using the Kruskal-Wallis and Mann-Whitney U tests. For biomechanical findings, group differences were analyzed using the one-way analysis of variance. The differences were evaluated at a level of significance of 0.05. All statistical analyses were performed using the SPSS 22.0 statistical software package.

## RESULTS

### Biomechanical findings

**Figure 1:**
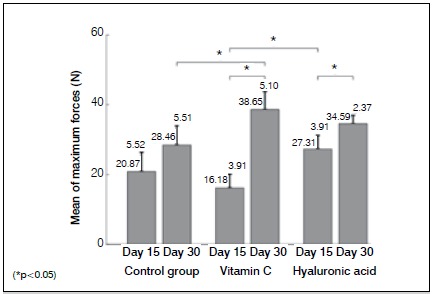
Mean of the maximum tensile force values of the tendons on Day 15 and Day 30.

In the biomechanical tests, the highest mean force value was observed in the HA group on Day 15, followed by the control and VC groups. (Figure 1) On Day 15, no significant difference was seen between the VC, HA, and control groups, with the exception of between the HA and VC groups (*p*=0.002). On Day 30, the VC group yielded the highest mean force value, followed by the HA and control groups. A significant difference was found between the control and VC groups (*p*=0.0162). In group comparisons between Day 15 and Day 30, remarkable improvement was seen in the VC group after Day 30 (*p*=0*.*00005). In addition, the maximum force value between Day 15 and Day 30 in the HA group increased significantly (*p*=0.0074).

### Histopathologic findings

Total semiquantitative histologic Movin and Bonar scores assessed on Day 15 and Day 30 for the HA, VC, and control groups are shown in Table 1 and Table 2, respectively. When compared to the HA and VC groups, the control group exhibited significantly higher total scores on Day 15 and Day 30 (*p*<0.05). However, no significant difference were seen in total Movin or Bonar scores between the HA and VC groups, on Day 15 or Day 30 (*p*>0.05).

**Table 1:** Semiquantitative Movin scores.

		Control group	Vitamin C	Hyaluronic acid	p
Day 15	Mean±SD	18.3±0.6	12.0±0.0	13.3±1.2	0.032
	Median (Min-Max)	18.0 (18.0-19.0)	12.0 (12.0-12.0)	14.0 (12.0-14.0)	
Day 30	Mean±SD	13.0±0.0	8.3±0.6	9.0±0.0	0.029
	Median (Min-Max)	13.0 (13.0-13.0)	8.0 (8.0-9.0)	9.0 (9.0-9.0)	
p for change between Day 15 and Day 30		0.051	0.034	0.034	

Kruskal-Wallis and Mann-Whitney U tests.

**Table 2:** Semiquantitative Bonar scores.

		Control group	Vitamin C	Hyaluronic acid	p
Day 15	Mean±SD	10.0±0.0	8.0±0.0	8.7±0.6	0.029
	Median (Min-Max)	10.0 (10.0-10.0)	8.0 (8.0-8.0)	9.0 (8.0-9.0)	
Day 30	Mean±SD	7.0±0.0	4.3±0.6	5.0±0.0	0.029
	Median (Min-Max)	7.0 (7.0-7.0)	4.0 (4.0-5.0)	5.0 (5.0-5.0)	
p for change between Day 15 and Day 30		0.100	0.025	0.025	

Kruskal-Wallis and Mann-Whitney U tests.

Compared with the baseline (Day 15), decreases in both Movin and Bonar scores were observed in all the groups on Day 30. While these decreases were not significant in the control group, the total scores were significantly improved on Day 30 in both the HA and VC groups (*p*<0.05).

## DISCUSSION

There is abundant literature on recovering maximum function after tendon repair, with particular focus on surgical techniques, application of exogenous agents to speed up healing, and the effect of comorbidities on tendon healing.^11^ With respect to tendon repair and tendon healing, this present study is the first to examine the therapeutic effects of HA and vitamin C in both biomechanical and histopathologic aspects. 

Derelioglu et al.^3^ examined the effect of HA on tendon healing and found no beneficial effects on tendon resistance. In our study, HA showed a strengthening effect on tendon resistance. 

Some experimental studies have reported notable reductions in postoperative adhesions after HA applications.^5^ ^,^ ^8^ ^,^ ^12^ Dabak et al.^8^ reported that rats treated with phospholipids and hyaluronic acid showed the lowest rates of adhesion, with no severe adhesions. In contrast, de Wit et al.^13^ found no significant difference in adhesion formation between HA- and saline-treated rabbit tendons.

Derelioglu et al.^3^ investigated the effects of HA, vitamin A, and vitamin E on tendon healing and adhesions and concluded that HA and vitamin A did not have a notable effect on the suppression of inflammatory response or the completion speed of the repair phase, while vitamin E was associated with a markedly reduced inflammatory response and more rapid achievement of mature collagen fibers and normal tendon structure. Greenwald et al.^14^ reported that vitamins A and E played a beneficial role in tendon healing through their action on differentiation, migration, and proliferation of fibroblasts. Foland et al.^15^ induced experimental tendinitis in horses and found that histopathologically, HA played a role in decreasing peritendinous fibrosis, fibroplasia, and anti-inflammatory response.

Gaughan et al.^12^ examined the effect of HA on tendon healing and adhesion formation in horses and found that HA reduced the number of inflammatory cells and the formation of new blood vessels, but did not have a notable effect on the development of normal tendon structure. In our histopathologic assessment, we also observed that high-dose vitamin C and HA had no effect on vascularity.

There are a large number of studies showing the benefits of HA associated with wound healing.^15^ Yagishita et al.^16^ found improved early tendon healing in rabbits treated with HA. De Wit et al.^13^ compared HA- and saline-injected rabbit digital flexor tendons and reported significantly increased breaking strength on biomechanical testing, and significantly accelerated tissue repair upon histopathologic examination in HA-treated rabbits.

During the early inflammatory phase of wound healing, high concentration of HA leads to increased infiltration and cell proliferation in the wound area. CD45 immunohistochemical staining showed that the cells in the repair area were leukocytes, most likely fibroblasts. These fibroblasts produce collagen fibers which improve repair and bridging and thus increase the tensile strength of the damaged tendon.^13^

Collagen contains two amino acids, hydroxyproline and hydroxylysine, which are essential for molecular stability. When these amino acids are synthesized, enzymes serve as a catalyst and oxygen, iron ions, alpha-ketoglutarate, and ascorbic acid are also required. As a result of the production of hydroxyproline-free collagen polypeptides, unstable collagen molecules are created. Ascorbic acid is a cofactor needed for the function of the prolyl hydroxylase enzyme involved at this stage.^17^

Although collagen biosynthesis and baseline collagen levels are inversely correlated with age, the positive effect of ascorbic acid on collagen synthesis is independent of age. In ascorbate-induced collagen synthesis, regulation of collagen gene expression is directly and specifically activated, which is enabled by both increased collagen gene copy number and the stability of procollagen mRNA. Another mechanism which stimulates collagen gene expression is the increased malondialdehyde level, a product of elevated lipid peroxidation.^18^

The need for ascorbic acid in prolyl and lysyl hydroxylase activity during collagen biosynthesis is well known, and the importance of this vitamin is increasingly pointed out for matrix proteoglycan synthesis. An in vitro experimental study found that the optimal level of ascorbic acid to maintain flexor tendons from adult animals in organ culture with 48-h media would be more than 50 micrograms/ml.^19^

Another experimental study investigated the effect of local vitamin C injection on tendon adhesion and found that this vitamin reduced adhesions of healing tendons.^20^ In our study, the role of vitamin C in reducing adhesions was not evaluated.

Omeroglu et al.^4^ administered high-dose vitamin C for rat Achilles tendon healing and evaluated the histopathologic results. In our study, we took their VC dose as a reference and found comparable results.

Unlike the studies in the literature, we not only conducted histopathologic analysis but also tested all the tendons biomechanically. The mean breaking forces were higher in the HA group on Day 15 (*p>0.05*) and in the VC group on Day 30 (*p<0.05*) than those of the control group. In-group comparisons indicated that the therapeutic effect of vitamin C on tendon healing was especially seen in the later period (*p=0.*00005). Moreover, the strength of the tendons in the HA group increased significantly from Day 15 to Day 30 (*p*<0.05). Our histopathologic findings showed a high correlation with the biomechanical results.

## CONCLUSIONS

Our study demonstrated that both vitamin C and hyaluronic acid had therapeutic effects on tendon healing, especially in the late phase of tendon repair. Further experimental studies may provide more conclusive data for the use of these two substances in Achilles tendon injuries.
